# Feasibility, effectiveness and safety of self-management in pulmonary rehabilitation: a study protocol using a hybrid type 1 effectiveness-implementation design

**DOI:** 10.3389/fresc.2023.1178823

**Published:** 2023-05-09

**Authors:** Ellen Ricke, Arie Dijkstra, Eric W. Bakker

**Affiliations:** ^1^ Department of Social Psychology, University of Groningen, Groningen, Netherlands; ^2^ Department of Epidemiology and Data Science, Division EBM, Academic Medical Centre, Amsterdam, Netherlands

**Keywords:** chronic obstructive pulmonary disease, pulmonary rehabilitation, self-management, feasibility, evaluation, prediction model

## Abstract

**Background:**

As population ageing accelerates worldwide, chronic diseases will place an increasing burden on society and healthcare systems. Self-management interventions may become a key strategy for addressing chronic disease burden and healthcare costs, also in pulmonary rehabilitation (PR). One of the challenges here is long-term adherence. Understanding the level of adherence to PR may help inform clinical decision-making to focus more on self-management and less on clinical supervision. For this reason, a prediction model (PATCH) was developed. The presented protocol concerns a study that aims 1. to evaluate the safety and effectiveness of self-management within pulmonary rehabilitation (PR) on health outcomes in patients with chronic obstructive pulmonary disease (COPD), 2. to evaluate the predictive validity of the PATCH tool, and 3. to evaluate feasibility and acceptability of self-management and the PATCH tool by patients and physiotherapists.

**Methods and analysis:**

This is a protocol of a hybrid type 1 effectiveness-implementation design, performed in primary physiotherapy practices in The Netherlands. The aim is to include 108 patients with COPD who have already followed PR for at least six weeks (maintenance stage of PR). According to the Dutch KNGF Guideline COPD, physiotherapists should reduce the number of supervised treatments after the maintenance phase and support self-management. In practice, this does not (always) happen. This protocol is based on implementing guideline advice: clinical supervision will be halved but patients are stimulated to engage in self-management by exercising unsupervised, leading to no change in the total planned exercise frequency. During the supervised sessions physiotherapists will assess and stimulate self-management. At baseline, and after 3, 6, 9 and 12 months, health outcomes (including adherence) will be evaluated as the primary outcome of this study. At each measurement, the physiotherapist will decide on the basis of individual scores whether the patient needs more clinical supervision or not. Secondary outcomes are the discriminatory power of the PATCH tool (can patients be correctly classified as adherent or non-adherent), and feasibility and acceptability of self-management and the PATCH tool by patients and physiotherapists. Questionnaires and semi-structured interviews will be used for assessment of the outcomes.

**Trial registration number**: METc 2023/074.

## 1. Introduction

As population ageing accelerates worldwide, chronic diseases will place an increasing burden on society and healthcare systems (1). Chronic diseases affect over 80% of people aged over 65 years in Europe, account for an estimated 77% of disease burden (2) and contribute to 70%–80% of healthcare costs (3). One of the chronic diseases with the highest burden of disease and cost is Chronic Obstructive Pulmonary Disease (COPD) (4). COPD is a heterogeneous lung condition characterized by chronic respiratory symptoms (dyspnea, cough, sputum production) due to abnormalities of the airways (bronchitis, bronchiolitis) and/or alveoli (emphysema) that cause persistent, often progressive, airflow obstruction (5).

Self-management interventions may become a key strategy for addressing chronic disease burden, contributing to the paradigm shift from a paternalistic model where patients are viewed as passive recipients of care, towards more equitable and collaborative models of healthcare provider-patient interaction (6). In doing so, self-management is seen as a possible solution to keep healthcare affordable. Self-management is defined as the partnering of healthcare providers with patients to support patients’ independent efforts to undertake long-term adherence to a preventive or therapeutic regimen that can improve functional status and health outcomes (7). There is evidence that COPD self-management interventions can improve quality of life at generally acceptable societal costs (8), and in some cases even result in short-term healthcare savings (9).

A core component of the management of COPD is pulmonary rehabilitation (PR) (10). It is often stated that PR is a cost-effective method of reducing dyspnea, increasing exercise capacity, and improving health-related quality of life in patients with COPD (8), and is recommended in national guidelines (10). According to the Dutch Koninklijk Nederlands Genootschap voor Fysiotherapie (KNGF) [Royal Dutch Society for Physiotherapy] Guideline COPD, self-management interventions should be implemented in PR treatment; physiotherapists should reduce the number of supervised treatments after the maintenance phase and support self-management (11). In practice, this does not (always) happen. One reason for this is that it appears that patients do not always adhere to recommendations, which negatively affects health outcomes (12, 13).

From a rehabilitation context, adherence has been defined as an “active, voluntary collaborative involvement of the patient in a mutually acceptable course of behavior to produce a desired preventive or therapeutic result” (14). Patients with COPD who are adherent to PR have better treatment outcomes (12, 15). However, in every situation in which patients have to take responsibility for their own treatment and supervised support is lowered, there is a substantial chance of non-adherence (12). This non-adherence could potentially reduce the effectiveness of PR, leading to lung attacks (16) and worsening of the disease and increased healthcare utilization (12, 15).

In sum, self-management regarding PR in patients with COPD could be a manner to reduce healthcare costs, but in doing so, the effectiveness of PR should not be compromised by non-adherence (17).

Tools to better understand and describe levels of adherence of patients with COPD to PR, may help inform clinical decision-making to focus more on self-management and less on clinical supervision. A prediction tool was therefore developed to estimate the likelihood that a patient will adhere to PR recommendations (18).

### 1.1. PATCH

The Predicting Adherence in paTients with CHronic diseases (PATCH) tool applies four predictors: patient's exercise intention, depression, patient-therapist relation (alliance), and Medical Research Council (MRC) dyspnea score (18). The model weighs and combines these predictors and gives the healthcare provider a probability that indicates the chance that the patient under evaluation will be adherent. Narrow validation showed good discrimination, calibration, and net benefit. To make PATCH more feasible for use in practice, the tool is available online: https://derzis.nu/Calculator/.

The effectiveness of phasing out supervised PR to be replaced by self-management regarding PR (guideline recommendation), nor the construct validity of the PATCH tool have been evaluated. First, it must be determined whether self-management is as effective and safe as fully supervised PR. Since adherence is an important precondition for considering self-management as an effective approach for a given patient, it should be assessed whether low adherence [as assessed with the Dutch version of the Rehabilitation Adherence Measure for Athletic Training (RAdMAT-NL)] indeed is associated with suboptimal self-management. Such a measurement design can simultaneous be used to assess the predictive validity of the PATCH tool. If self-management is effective and safe, it can be explored how it can be implemented in clinical practice. Since implementation of new routines in healthcare is challenging and complex (19), it is wise to anticipate feasibility. There may be a variety of reasons (characteristics of the PATCH tool, characteristics of the physiotherapist, characteristics of the patient) that lead to neither the promotion of self-management, nor the PATCH tool being adopted in clinical practice.

### 1.2. Objectives

The primary aim of this study is to evaluate the safety and effectiveness of self-management within pulmonary rehabilitation (PR) according to guideline recommendations as a partial replacement of supervised PR in patients with COPD who have already been participating in PR for at least six weeks, in a primary physiotherapy practice in the Netherlands. The secondary aim is to evaluate the predictive validity of the PATCH tool in this same cohort of patients over 12 months. Finally, a third aim is to evaluate perceived feasibility and acceptability of self-management and the PATCH tool by patients and physiotherapists. The purpose of this paper is to describe the rationale and design of the study.

## 2. Methods

### 2.1. Study design and setting

This is a protocol of a hybrid type 1 effectiveness-implementation design that combines an effectiveness study with an implementation study (20). A prospective cohort study of 12 months will evaluate the safety and effectiveness of self-management within PR according to guideline recommendations as a partial replacement of supervised PR on health outcomes (i.e., adherence, exercise capacity, MRC dyspnea score, health related quality of life, disease burden, lung attacks), and the predictive validity of the PATCH tool. In addition, implementation will be evaluated in terms of perceived feasibility and acceptability of self-management and the PATCH tool by patients and physiotherapists through questionnaires and semi-structured interviews.

**Figure 1:**
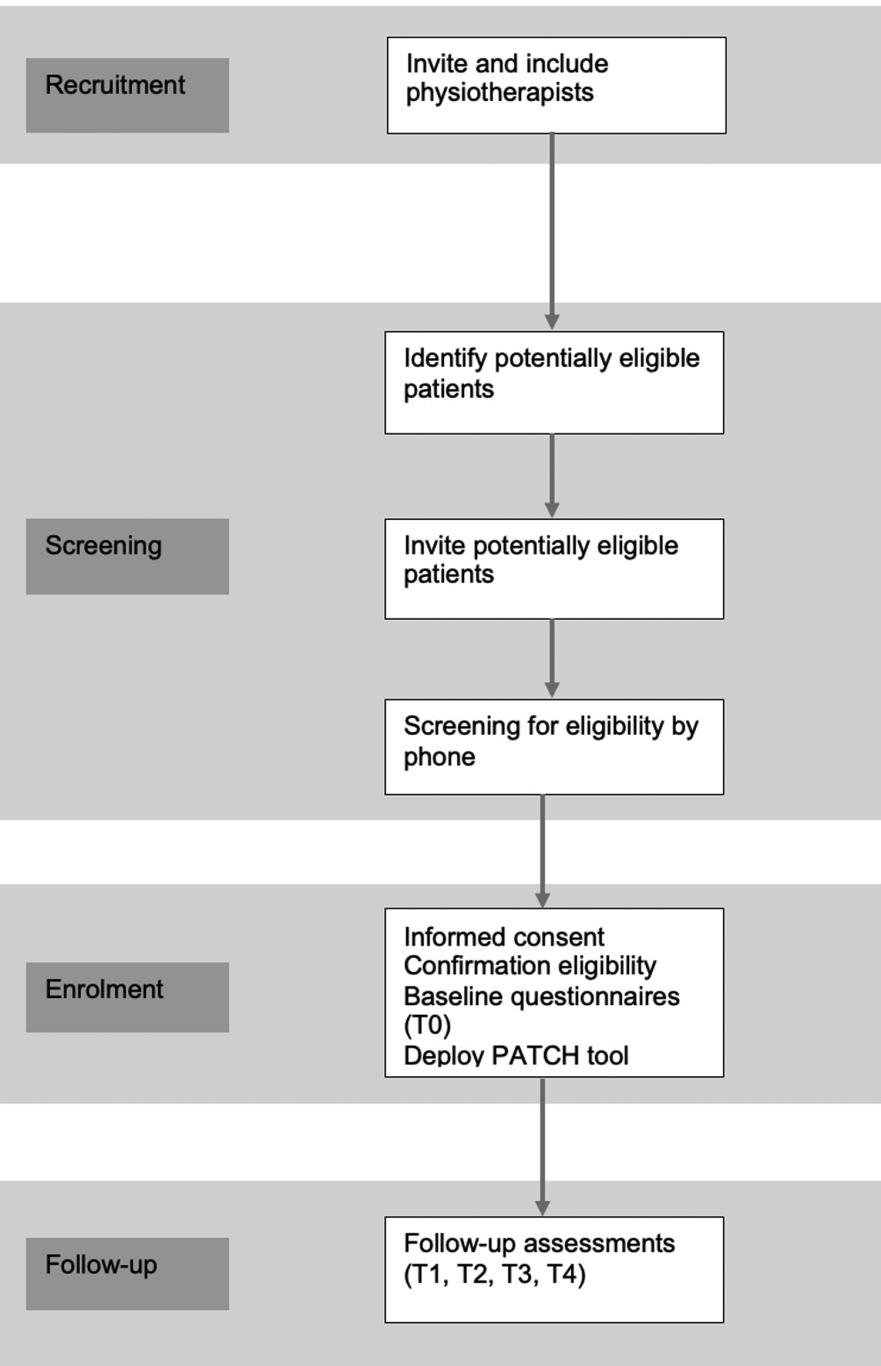
Study flow diagram. T1, 3 months, T2, 6 months, T3, 9 months, and T4, 12 months.

The study will be conducted in primary physiotherapy practices in the Netherlands. Physiotherapists will be eligible if their practice has access to a computer and internet. Eligible physiotherapists who provide informed consent will be enrolled into the study. Patients with COPD from the participating practices who indicate an interest in participation in the study, will be screened for eligibility by the researcher, and have follow-up measurements at 3, 6, 9 and 12 months from baseline. Physiotherapists and patients are recruited throughout the Netherlands. The design of the study and flow of practices and patients is shown in Figure 1. The protocol is reported according to the Standard Protocol Items: Recommendations for Interventional Trials (SPIRIT) guidelines (21) (Supplementary Additional File S1).

### 2.2. Physiotherapist recruitment

Physiotherapists will be contacted through Chronic CareNet, a Dutch quality network of more than 3,500 primary care providers (physiotherapists, exercise therapists and dieticians) who specialize in treating patients with non-communicable chronic conditions, including lung disease (22). By recruiting physiotherapists through Chronic CareNet, an attempt is made to recruit the most homogeneous group of physiotherapists possible; that is, they must all meet the quality standards, i.e., in terms of their own education, the use of clinimetrics, the use of guidelines. Physiotherapists will be invited to participate via an e-mail containing study information and an information folder about the PATCH tool. Physiotherapists will be followed up via telephone contact and e-mail.

### 2.3. Patient recruitment

Patients will be recruited from enrolled primary physiotherapy practices. By inviting patients who receive PR from physiotherapists who are members of Chronic CareNet, and who meet the inclusion and exclusion criteria, a study population is constituted that represents as closely as possible the “average” patient with COPD in the Netherlands. E-mails or invitation letters with an expression of interest form, and a reply envelope will be sent on behalf of the physiotherapist to all eligible patients. Non-responders will be sent a reminder e-mail/letter after 2–3 weeks. Furthermore, physiotherapists will be asked to remind the non-responders when they are visiting the physiotherapy practice.

### 2.4. Inclusion and exclusion criteria

On expression of interest, all potentially eligible patients will be screened by the researcher by telephone for eligibility. Patients (≥18 years) with COPD, with airflow limitation stage GOLD II-IV, who are in the maintenance phase (therapeutic treatment) of rehabilitation (have had at least 6 weeks of PR) (11), and having rehabilitation sessions once a week, will be eligible for inclusion. Also, patients must provide written informed consent. They will be informed that when they take part in the study, they will still be free to withdraw at any time, without giving a reason; their decision will not affect the usual care they receive. The exclusion criteria are home-based rehabilitation, patients eligible for maintenance treatment (11), and insufficient mastery of the Dutch language to complete the questionnaires.

### 2.5. Self-management

All patients receive usual care according to the Dutch KNGF Guideline COPD (11). This includes determining the training frequency, intensity and duration of an exercise according to this guideline. With the introduction of more self-management, clinically supervised exercise sessions are reduced by half at T0, and patients are instructed to complete additional unsupervised exercise with the intention that overall exercise frequency remains unchanged. In practice, this means that the patients, who currently receive clinical supervision once a week, will receive clinical supervision every other week and be instructed to complete an unsupervised exercise program in the remaining weeks. During the supervised treatments physiotherapists will be asked to pay attention to self-management, including discussion of the impact of COPD and lung attacks on physical functioning. Further, they are instructed to provide education on the patient's own role in treatment and coping strategies for managing their condition according to the guideline recommendations (11).

Based on shared decision-making, the physiotherapist and patient agree on a desired form of unsupervised exercise (appropriate to the context of the patient). If the physiotherapy practice has the facility, the patient may choose to exercise independently at the physiotherapy practice. The patient may also choose to exercise independently at home or use regular sports and exercise services.

**Figure 2:**
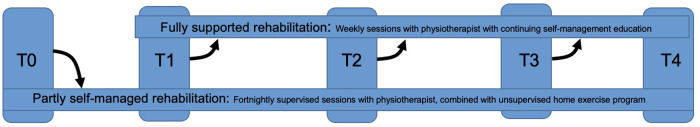
Process of supervision over time. At T0, all patients start with reduced clinical supervision (partly self-management of rehabilitation). At each measurement moment (T1–T3), a decision is made whether the patient can remain in reduced clinical supervision, or move to full clinical supervision.

For 12 months, the physiotherapist will monitor the patient's primary COPD-related health outcomes and patient's adherence. Follow-up moments will occur every three months [consistent with the KNGF guideline COPD (11)]. These follow-up moments are important in order to adjust the intensity of clinical supervision if necessary; patients should not have any decline in health-related outcome measures (including adherence). If at any follow-up moment there is a decline in any of the health-related outcome measures, the patient will receive full clinical supervision again (Figure 2). It is expected that patients who are adherent at a follow-up (RAdMAT-NL score ≥54), are capable of more self-management while maintaining stable health outcomes over the next three months. Patients who are non-adherent (RAdMAT-NL score <54) are expected to have more difficulty with self-management and experience a decline in health outcomes. Given this expectation, patients who show a decline in adherence scores will receive full clinical supervision to prevent their health outcomes from declining.

### 2.6. Data collection and follow-up

Each patient and physiotherapist will provide data through questionnaires at baseline, 3 months, 6, 9 and 12 months after inclusion (T0–T4) (Table 1). A subgroup of physiotherapists and patients will be invited to take part in a semi-structured interview in order to provide their perspectives on self-management and the PATCH tool.

**Table 1:** Overview of measurements.

	Run-in	Intervention				
Contact moment	T-1	T0	T1	T2	T3	T4
Physiotherapist characteristics		X				
Informed consent	X					
Eligibility assessment	X					
Probability of adherence (PATCH)		X				
Patient assessment		X				
Exercise adherence		X	X	X	X	X
Perceived overall adherence		X	X	X	X	X
Exercise capacity		X	X	X	X	X
Health related quality of life		X	X	X	X	X
Disease burden		X	X	X	X	X
Perceived dyspnea		X	X	X	X	X
Lung attacks		X	X	X	X	X
Number of scheduled appointments		X	X	X	X	X
Implementation assessment						X

T-1, run-in period; T0, baseline; T1, 3 months; T2, 6 months; T3, 9 months; T4, 12 months.

All questionnaires for both patients and physiotherapists will be prepared in Qualtrics (online survey software) (23). The questionnaires will be sent by the researcher from Qualtrics to the participants at the different measurement moments. Answers to the questionnaires will automatically be collected in Qualtrics. If a questionnaire is not completed after two weeks, an automatic reminder will be sent. The researcher enters the responses of patients who complete a paper questionnaire into Qualtrics to obtain a complete data file of all participants. Prior to data analysis, the raw data file will be checked by the researcher for any input errors. If input errors are found, the researcher will contact the respective participant (patient and/or physiotherapist), if possible. In this way, the raw data file is made technically correct and consistent. Data will be anonymized by deleting the e-mail addresses and adding a PID number. Based on the PID number measurements of all measurement moments can be merged.

#### 2.6.1. Baseline (T0) questionnaires

Baseline data from the participating physiotherapists will be collected at the time of enrolment (T0): gender (male/female), entry-level degree, post graduate qualification (yes/no), and professional experience in a primary physiotherapy practice (years).

Baseline data from the patients will be collected after signing informed consent and inclusion (T0): age (years), gender (male/female), education (coded into low/middle/higher), smoking status (never smoked/quit smoking/still smoking), health related quality of life (1–5), and disease burden (0–60) (all provided by the patient). The physiotherapist will provide information on the characteristics of the disease (T0): classification of severity of airflow limitation [Global Initiative for Chronic Obstructive Lung Disease (GOLD) classification] (GOLD II/III/IV), degree of baseline functional disability due to dyspnea (MRC dyspnea score) (0/1/2/3/4/5), duration of COPD since diagnosis (years), and duration of physiotherapy (years).

#### 2.6.2. Study measures

##### 2.6.2.1. Exercise capacity

Exercise capacity will be assessed by the physiotherapist at every measurement moment (from T0–T4) using the 6-minute walk distance (6MWD). The change in 6MWD is a potential patient-centered outcome measure for therapies aimed at maintaining/improving exercise capacity (24). Patients have to try to cover as much distance as possible in six minutes; the physiotherapist measures the walking distance (25).

##### 2.6.2.2. Health related quality of life

Health related quality of life will be assessed by the PROMIS Global02 (26) at each measurement moment (T0–T4) by the patient. The PROMIS Global02 is derived from the Overall Health V1.2. scale and asks about the patient's perceived quality of life. The exact question is, “Overall, how do you feel about your quality of life?” The PROMIS Global02 uses a five-point rating scale (5 = excellent, 4 = very good, 3 = good, 2 = fair, 1 = poor). A higher score indicates better health related quality of life (26).

##### 2.6.2.3. Disease burden

Specific COPD disease burden will be reported by the patient at each measurement moment (T0–T4) using the Clinical COPD Questionnaire (CCQ). The questionnaire consists of ten items divided into three domains: symptoms, functional state and mental state. Individual items within the CCQ are equally weighted, and the mean item score will be used as the total scale score. Additionally, it is possible to calculate the scores on each of the three domains separately. The total CCQ score, and the score on each of the three domains, varies between 0 (very good health status) to 6 (extremely poor health status) (27).

##### 2.6.2.4. Perceived dyspnea

Patients will report their perceived dyspnea at each measurement moment (T0–T4) using the MRC dyspnea scale. The MRC scale is a practical and validated instrument to score the degree of dyspnea as experienced by a patient with COPD (28). The list consists of 5 items in which the patient can indicate their own level of limitation: grade 1 “I only get breathless with strenuous exercise”; grade 2, “I get short of breath when hurrying on the level or up a slight hill”; grade 3, “I walk slower than people of the same age on the level because of breathlessness or have to stop for breath when walking at my own pace on the level”; grade 4, “I stop for breath after walking 100 yards or after a few minutes on the level”; grade 5, “I am too breathless to leave the house” (28).

##### 2.6.2.5. Lung attacks

Patients will report at each measurement moment whether they have had a lung attack (16) in the past three months, and if so, how many. Here, a lung attack is defined as a sudden worsening of lung symptoms for which the general practitioner or pulmonologist had to prescribe antibiotics or prednisolone (16, 29).

##### 2.6.2.6. Number of scheduled rehabilitation appointments

At baseline (T0), physiotherapists will indicate how many appointments their patient had in the previous three months. If the patient has PR for less than three months, the number of appointments and duration of PR to that point will be noted. At each follow-up moment (T1–T4), physiotherapists will again record how many appointments their patient had during that period.

##### 2.6.2.7. Exercise adherence

Exercise adherence will be assessed by the Dutch version of the Rehabilitation Adherence Measure for Athletic Training (RAdMAT-NL). The RAdMAT-NL has good psychometric properties with an internal consistency reliability of *α* = 0.91 (30). The RAdMAT-NL is a 16-item questionnaire that uses a 4-point rating scale (never = 1, occasionally = 2, often = 3, always = 4) to evaluate clinic-based adherence (30). The RAdMAT-NL consists of 3 subscales: Attendance/participation (items 1–5, range 5–20 points), Communication (items 6–8, range 3–12 points), and Attitude/effort (items 9–16, range 8–32 points). The total scale range is 16–64, a higher score indicates a higher degree of adherence. According to the American College of Sports Medicine guidelines, a score of at least 85% must be achieved to be adherent to the rehabilitation program (31). This means, a minimum total score of 54 or higher must be achieved on the RAdMAT-NL to be adherent. The RAdMAT-NL will be completed at baseline (T0) and all follow-up moments (T1–T4) by the physiotherapist, independent of the patient and not in their presence.

##### 2.6.2.8. Perceived overall adherence

Asking a single direct question is a strategy to assess patients' adherence (32).

With a single question, patients will be asked to rate their perceived overall adherence (home-based and clinic-based adherence) considering the extent to which they succeed to attend appointments, to perform prescribed exercises, and the extent to which they ask questions and give feedback about their rehabilitation. Patients will report their perceived overall adherence at each measurement moment (T0–T4) on a five-point rating scale (did not succeed at all = 1, did succeed for less than half = 2, did succeed for about half = 3, did succeed for more than half = 4, totally succeeded = 5).

##### 2.6.2.9. Probability of adherence

The probability of adherence over 12 months will be assessed by the physiotherapist using the PATCH tool at baseline (T0). The PATCH tool integrates predictors (intention, depression, MRC-score, alliance), and is intended for use in the population of COPD patients following PR for at least one month (18). Internal validation showed good discrimination, calibration, and net benefit. The calculator provides a probability output that indicates the chance that the particular patient under evaluation is adherent (probability score ≥ 53.5%) (18). For example, a percentage of 75% indicates that there is a 75% chance that the patient is adherent (according to the criterion of engaging in at least of 85% of the prescribed exercise).

##### 2.6.2.10. Implementation

Implementation outcomes will be defined by the Reach, Effectiveness, Adoption, Implementation and Maintenance (RE-AIM) framework, a framework designed to evaluate multiple dimensions of evidence-based intervention implementation in order to determine the public health impact and to shape scale-up processes (33). Implementation outcome measures are specified by each component of RE-AIM and are summarized in Table 2. Through these components, the impact of innovations can be assessed at both the individual (i.e., end-user; reach, effectiveness, maintenance) and organizational (i.e., delivery agent; adoption, implementation, maintenance) levels (34). These implementation outcomes will be assessed at T4 by the researcher during interviews with physiotherapists and patients.

**Table 2:** Key components of the self-management and PATCH tool intervention.

RE-AIM dimension	Implementation question	Outcome measure(s)
Reach	Does the information on the availability of the intervention (PATCH tool and self-management) reach the target population (physiotherapists in primary care)?	Number, percentage and representativeness of eligible physiotherapists, who heard about the intervention. Expansion with qualitative questions: What are your expectations of the intervention?
Effectiveness	Does the intervention (PATCH tool and self-management) accomplish its goals?	Relative change in number of clinical appointments over time. Number, percentage of patients with stable health outcomes. Expansion with qualitative questions: What are the conditions and mechanisms that lead to effectiveness?
Adoption	To what extent are those targeted to deliver the intervention (physiotherapists) actually using it?	The number of physiotherapists who are using the intervention as protocolled. Expansion with qualitative questions: What affects physiotherapists adoption?
Implementation	Was the intervention implemented as intended? (fidelity) How consistent was delivery across settings and staff?	Number, percentage of physiotherapists providing measures of all (four) follow-up moments. Number of patients who have been exposed to the intervention. Expansion with qualitative questions: What were the modifications to the intervention and why did they occur? What were the barriers to fidelity? What are the contextual factors and processes underlying barriers to implementation and how can they be addressed?
Maintenance	To what extent did the intervention become part of routine organizational practices? To what extent are professional organizations interested to support the use of the intervention?	Proportion of physiotherapists who offer self-management at the beginning and after the end of the study period. Proportion of physiotherapists using the PATCH tool at the beginning and after the end of the study period. Expansion with qualitative questions: Do the Lung Foundation and the KNGF support the intervention? What modifications are made after the study? What are the barriers to maintaining the program?

#### 2.6.3. Semi-structured interviews

Interviews with physiotherapists and patients will be conducted by a trained researcher at T4. An interpretivist, constructivist, approach will be used to gain an in-depth understanding of participant's views of more self-management and the PATCH tool (35). Interpretivism is an approach to social science that asserts that understanding the beliefs, motivations, and reasoning of individuals in a social situation is essential to decoding the meaning of the data that can be collected around a phenomenon (36). Table 2 will be used as a semi-structured interview topic guide to address the feasibility and acceptability of self-management and the PATCH tool by patients and physiotherapists. All interviews will be audio-recorded on a digital voice recorder, transcribed verbatim in Microsoft Word using Express Scribe 11 (37), and then anonymized.

### 2.7. Sample size

#### 2.7.1. Sample size quantitative data

The power calculation is based on the primary outcome most important to the patient: health related quality of life over 12 months, as measured by the PROMIS Global02. A patient reported outcome (PRO) was chosen because it is increasingly important to capture the patients' perspective of treatment effectiveness (38). In addition, for sample size calculation, it is important to choose the most conservative outcome measure, as this requires the most participants (39); in this case also health related quality of life.

The effect is expressed as the absolute difference in mean health related quality of life within each patient over time. In the present design in which supervised PR is scaled down and replaced by self-management, it is especially important to be able to detect even very small declines in quality of life. Therefore, an effect size of *d* = 0.2 (difference is very small) is chosen.

To calculate the sample size required to detect a very small, not necessarily clinically relevant, but undesirable effect, an *a priori* analysis is performed using G power. With an F test as test family, an ANOVA repeated measures within factors as statistical test, a given *α*, power and effect size (*α* = 0.05 and *β* = 0.95, effect size = 0.2), and number of measurements = 5 (T0–T4), a sample size of 48 patients is needed. Loss to follow-up must be taken into account; patients leaving the study early. In a previous study of patients with COPD who received PR (18), the drop-out rate was 6.1%, and a drop-out rate of 20% is still considered normal. Therefore, an average is taken and a drop-out rate of 13% is taken into account, meaning 54 patients have to be included.

To make it also possible to perform a subgroup analysis between adherent and non-adherent patients, as defined at T0, the estimated sample size must be doubled; a total sample size of 108 patients is therefore needed.

#### 2.7.2. Sample size qualitative data

Purposive sampling will be used to maximize variation, identifying adherent and non-adherent patients, and physiotherapists who want to continue the use of the PATCH tool and those who do not. Potential participants will be invited by the researcher. A purposive sampling approach will be used to ensure that not a sample of extremes will be gathered or that the perspectives of one group of individuals are overrepresented (40), for example, those who are non-adherent. We aim for 10 participants per group (*n* = 40), which is typically a sufficient number to reach thematic saturation, the point when new themes are no longer emerging from the data (40).

### 2.8. Statistical analysis

#### 2.8.1. Analysis quantitative data

Data will be analyzed using R version 4.0.3 (41). First, for missing data, the amount of missingness for each variable will be calculated; the difference between the sample size and the number of useable observations. Second, Fisher exact tests will be used to analyze differences in baseline characteristics between patients with missing and complete data. When data are missing at random, multiple imputation will be used to create and analyze five multiple imputed datasets. Incomplete variables will be imputed under fully conditional specification, using the default settings of the mice 3.0 package (42). The parameters of substantive interest will be estimated in each imputed dataset separately, and combined using Rubin's rules.

Data will be screened for outliers and tested for normal distribution. Descriptive statistics will be used to summarize the baseline characteristics of the physiotherapists, and baseline demographic and clinical characteristics of the patients. Variables will be expressed in percentages, in the mean ± standard deviation (SD) or as the median with interquartile range (IQR), depending on which is appropriate.

The outcomes are repeatedly measured in clusters. So, to evaluate changes in health outcomes, clinical supervision, and adherence over time, a multilevel linear mixed-model analysis will be performed (43). The model will include health related quality of life per patient from baseline to T4 as the dependent variable. The mixed model will include a random intercept per physiotherapy practice. A correlation structure will be chosen for the repeated measurements on the level of patients by selecting the best fitting variance–covariance matrix. A similar approach will be used for the outcome adherence, number of scheduled appointments, exercise capacity, MRC-score, disease burden, and lung attacks.

The discriminatory ability of the PATCH tool (the validity) will be determined by comparing the proportion of patients correctly classified as adherent or non-adherent with the actual classification measured by the RAdMAT-NL, quantified as the area under the receiver operating characteristic curve (AUROC). Using the AUROC, the threshold of the PATCH tool will be determined. In a prospective study in COPD patients in PR, the current PATCH tool had a sensitivity of 67.9% and specificity of 75.9% (18).

For all tests, *p*-values <0.05 will be considered statistically significant.

#### 2.8.2. Analysis qualitative data

The semi-structured interviews will be analyzed using N-vivo14 (44). As a strategy for analysis systematic text condensation (STC) will be used (45). STC is a modification of Giorgi's phenomenological analysis and encompasses thematic analysis of meaning and content of data across cases (45). Finally, the results of the two-level mixed model will be integrated with the results of the thematic analysis using joint displays (46). Joint display brings qualitative and quantitative data together through a visual means to “draw out new insights beyond the information gained from the separate quantitative and qualitative results” (46).

## 3. Data management

All data will be processed anonymously, according to the guidelines of the University of Groningen (RUG). The data will be stored digitally on the highly secured server of the RUG. All local databases are secured with password-protected access systems. All records that contain names or other personal identifiers, such as informed consent forms are stored separately and pseudonymized from study records identified by code number (47).

The data may be used for a scientific publication and for educational purposes, but it will never be traceable to the individual patient. Patients can always ask to withdraw data until personal identifiers have been removed.

## 4. Ethics approval

This study protocol is registered with the number METc 2023/074. The METc UMCG has concluded that the study is not clinical research with human subjects as meant in the Medical Research Involving Human Subjects Act (WMO).

## 5. Discussion

This study protocol details the evaluation of the safety and effectiveness of COPD-patient self-management regarding pulmonary rehabilitation according to guideline recommendations in primary physiotherapy practices in the Netherlands. With a follow-up of 12 months, the study will provide insight in the relationship between self-management and adherence, and the COPD-specific health outcomes exercise capacity, disease burden, health related quality of life, MRC dyspnea score, and lung attacks, over the medium term.

In addition, this study also evaluates the predictive validity of the Predicting Adherence in paTients with CHronic diseases (PATCH) tool, and the implementation outcomes feasibility and acceptability of self-management and the PATCH tool.

It has become increasingly acknowledged that health innovations (including providing healthcare in an alternative manner) should be evaluated in real-life conditions; this study resembles real-life as much as possible. The inclusion is inclusive and patients and the participating physiotherapists do not receive prescribed instructions about where unsupervised exercise should take place, and they are expected to follow the commonly used Dutch KNGF Guideline COPD. This allows patients and physiotherapist to decide on the setting and type of exercise that best suits the patient's needs and context based on shared-decision making.

### 5.1. Research design

As research design a prospective treatment cohort was chosen, because (1) no intervention will be implemented, the reduction (in this case 50%) of clinical supervision is described in the KNGF Guideline COPD and can be considered as usual care (therefore no RCT); (2) patients will be followed in time to evaluate whether there are changes in treatment outcomes (therefore prospective); (3) only patients who receive clinical supervision, as opposed to patients who already exercise independently, will be included (therefore treatment cohort). A disadvantage of this design is that only the association between self-management and health outcomes, and not causation, can be inferred from the results of the study (48). However, by following patients over time and measuring various variables, it can be determined which patients are at greater risk for poorer health outcomes. Furthermore, a mixed-method approach was chosen because experiences can vary from person to person. Therefore, qualitative measures are added to the quantitative measures; participants should be able to provide an explanation in their own words, e.g., why they think self-management is or is not effective (49). The function of this mixed-method approach is using the qualitative data to explain the results of the analysis of the quantitative data. Finally, this study does not focus only on outcomes. Instead, the whole process of implementation is taking into account. The study is based on a hybrid type 1 effectiveness-implementation design, focusing on testing the effects of a clinical intervention on relevant outcomes while observing and gathering information on implementation, in order to more rapidly move interventions from effectiveness testing through implementation to public health impact (20). This design was chosen as the effectiveness of more self-management (more unsupervised exercise) regarding PR has not yet been demonstrated in primary physiotherapy settings in the Netherlands and little information exists on implementation of unsupervised exercise in primary care. An additional advantage of this design is that the predictive validity and implementation of the PATCH tool can be evaluated in the same cohort, which reduces the burden on physiotherapists and patients and saves time and money.

### 5.2. Self-management and follow-up

The KNGF Guideline COPD includes a section on supervision by the physiotherapist: “In the intensive treatment phase, strive to achieve the treatment goal. Strive in the phase-out phase to maintain the treatment goal and in the maintenance phase of the therapeutic treatment to transition to regular sports and exercise activities” (11). For patients who are considered for maintenance treatment, a treatment frequency of once a week is advised until the patient is able to exercise independently (11). Based on this guideline, it was chosen to halve clinical supervision. As an inclusion criterion, it was chosen to include only patients who receive PR once a week. This choice was made because training theory indicates that exercising once a week is considered a maintenance treatment (11). When patients go from twice-weekly to once-weekly supervised exercise, it may not be possible to measure a difference in health outcomes due to maintenance treatment. However, supervised exercise every other week will be able to show significant differences in treatment outcomes, (not only due to less exercise, but possibly also due to change in social aspect). The guideline is also followed in terms of follow-up moments. In practice, it turns out that it is not feasible for every physiotherapist to repeat all measurements at every evaluation moment. Possibly, if the discriminatory ability of the PATCH tool turns out to be good, the PATCH tool could be used to determine which patients should be evaluated every three months and which less frequently. Finally, patients are included who are in the maintenance phase of PR because they need to reduce the treatment frequency of the intensive treatment phase. So, the choice of participants of this study is also based on the KNGF Guideline COPD.

In terms of risks and (unintended) effects, for participation in this study no severe or unexpected adverse events will be expected, because of the regular follow-ups. Nevertheless, it has to be kept in mind that patients could feel uncomfortable with the change in their supervision and the focus on self-management.

### 5.3. Outcomes

The outcomes of this multicenter study will add to the evidence on the effectiveness of partially replacing supervised exercise with unsupervised exercise in a PR program, and of initially using an adherence prediction tool to identify patients who are adherent and who are non-adherent. Because of the hybrid type 1 effectiveness-implementation design, it will give important insights in the acceptability of unsupervised exercise and the use of an exercise adherence prediction model in clinical practice from the perspective of patients and physiotherapists. If unsupervised exercise is feasible and acceptable, this study will inform the implementation of more self-management in clinical practice in other physiotherapy practices.

So far, the optimal cut-off value for the PATCH tool is used. However, the PATCH tool applies a statistical prediction model and therefore does not take into account other individual factors, other than those of the prediction model, that may affect adherence, such as multimorbidity, for example. Hence, it is important to evaluate whether the cut-off value needs to be adjusted. With the results of this study, including clinical relevance, the cut-off value of the PATCH tool may be changed. For example, no patients should be classified as false positive (a patient is classified as adherent but he or she is not). In that case, a patient would incorrectly receive less clinical supervision. When the PATCH tool identifies patients effectively as adherent or non-adherent, the study will inform the implementation of the PATCH tool in other physiotherapy practices. The PATCH tool could then not only be used to determine treatment frequency, but could then potentially be used to reduce the registration burden (fewer evaluation moments).
