# Supplementary material for: Feasibility, effectiveness and safety of self-management in pulmonary rehabilitation: a study protocol using a hybrid type 1 effectiveness-implementation design
Source: Front Rehabil Sci. 2023 May 9;4:1178823. doi: 10.3389/fresc.2023.1178823 (PMC10203526; doi:10.3389/fresc.2023.1178823)
Supplement: Supplementary file 1 [file Datasheet1.docx]

**Addendum ‘Feasibility, effectiveness and safety of self-management in pulmonary rehabilitation: a study protocol using a hybrid type 1 effectiveness-implementation design’**

**Contents**

Additional file 1: SPIRIT checklist

Additional file 2: Prognostic model PATCH

**Additional file 1: SPIRIT checklist**


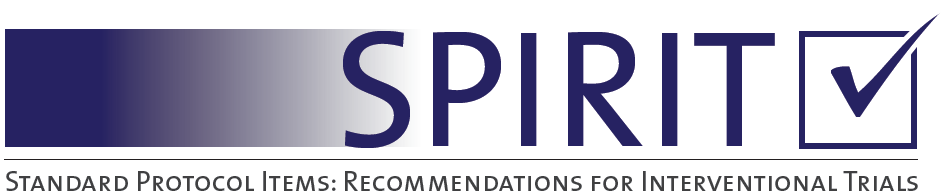


SPIRIT 2013 Checklist: Recommended items to address in a clinical trial protocol and related documents*

| Section/item | ItemNo | Description |
| --- | --- | --- |
| **Administrative information** | | |
| Title | 1 | Descriptive title identifying the study design, population, interventions, and, if applicable, trial acronym |
| Trial registration | 2a | Trial identifier and registry name. If not yet registered, name of intended registry |
|  | 2b | All items from the World Health Organization Trial Registration Data Set |
| Protocol version | 3 | Date and version identifier |
| Funding | 4 | Sources and types of financial, material, and other support |
| Roles and responsibilities | 5a | Names, affiliations, and roles of protocol contributors |
|  | 5b | Name and contact information for the trial sponsor |
|  | 5c | Role of study sponsor and funders, if any, in study design; collection, management, analysis, and interpretation of data; writing of the report; and the decision to submit the report for publication, including whether they will have ultimate authority over any of these activities |
|  | 5d | Composition, roles, and responsibilities of the coordinating centre, steering committee, endpoint adjudication committee, data management team, and other individuals or groups overseeing the trial, if applicable (see Item 21a for data monitoring committee) |
| Introduction |  |  |
| Background and rationale | 6a | Description of research question and justification for undertaking the trial, including summary of relevant studies (published and unpublished) examining benefits and harms for each intervention |
|  | 6b | Explanation for choice of comparators |
| Objectives | 7 | Specific objectives or hypotheses |
| Trial design | 8 | Description of trial design including type of trial (eg, parallel group, crossover, factorial, single group), allocation ratio, and framework (eg, superiority, equivalence, noninferiority, exploratory) |
| Methods: Participants, interventions, and outcomes | | |
| Study setting | 9 | Description of study settings (eg, community clinic, academic hospital) and list of countries where data will be collected. Reference to where list of study sites can be obtained |
| Eligibility criteria | 10 | Inclusion and exclusion criteria for participants. If applicable, eligibility criteria for study centres and individuals who will perform the interventions (eg, surgeons, psychotherapists) |
| Interventions | 11a | Interventions for each group with sufficient detail to allow replication, including how and when they will be administered |
|  | 11b | Criteria for discontinuing or modifying allocated interventions for a given trial participant (eg, drug dose change in response to harms, participant request, or improving/worsening disease) |
|  | 11c | Strategies to improve adherence to intervention protocols, and any procedures for monitoring adherence (eg, drug tablet return, laboratory tests) |
|  | 11d | Relevant concomitant care and interventions that are permitted or prohibited during the trial |
| Outcomes | 12 | Primary, secondary, and other outcomes, including the specific measurement variable (eg, systolic blood pressure), analysis metric (eg, change from baseline, final value, time to event), method of aggregation (eg, median, proportion), and time point for each outcome. Explanation of the clinical relevance of chosen efficacy and harm outcomes is strongly recommended |
| Participant timeline | 13 | Time schedule of enrolment, interventions (including any run-ins and washouts), assessments, and visits for participants. A schematic diagram is highly recommended (see Figure) |
| Sample size | 14 | Estimated number of participants needed to achieve study objectives and how it was determined, including clinical and statistical assumptions supporting any sample size calculations |
| Recruitment | 15 | Strategies for achieving adequate participant enrolment to reach target sample size |
| **Methods: Assignment of interventions (for controlled trials)** | | |
| Allocation: |  |  |
| Sequence generation | 16a | Method of generating the allocation sequence (eg, computer-generated random numbers), and list of any factors for stratification. To reduce predictability of a random sequence, details of any planned restriction (eg, blocking) should be provided in a separate document that is unavailable to those who enrol participants or assign interventions |
| Allocation concealment mechanism | 16b | Mechanism of implementing the allocation sequence (eg, central telephone; sequentially numbered, opaque, sealed envelopes), describing any steps to conceal the sequence until interventions are assigned |
| Implementation | 16c | Who will generate the allocation sequence, who will enrol participants, and who will assign participants to interventions |
| Blinding (masking) | 17a | Who will be blinded after assignment to interventions (eg, trial participants, care providers, outcome assessors, data analysts), and how |
|  | 17b | If blinded, circumstances under which unblinding is permissible, and procedure for revealing a participant’s allocated intervention during the trial |
| **Methods: Data collection, management, and analysis** | | |
| Data collection methods | 18a | Plans for assessment and collection of outcome, baseline, and other trial data, including any related processes to promote data quality (eg, duplicate measurements, training of assessors) and a description of study instruments (eg, questionnaires, laboratory tests) along with their reliability and validity, if known. Reference to where data collection forms can be found, if not in the protocol |
|  | 18b | Plans to promote participant retention and complete follow-up, including list of any outcome data to be collected for participants who discontinue or deviate from intervention protocols |
| Data management | 19 | Plans for data entry, coding, security, and storage, including any related processes to promote data quality (eg, double data entry; range checks for data values). Reference to where details of data management procedures can be found, if not in the protocol |
| Statistical methods | 20a | Statistical methods for analysing primary and secondary outcomes. Reference to where other details of the statistical analysis plan can be found, if not in the protocol |
|  | 20b | Methods for any additional analyses (eg, subgroup and adjusted analyses) |
|  | 20c | Definition of analysis population relating to protocol non-adherence (eg, as randomised analysis), and any statistical methods to handle missing data (eg, multiple imputation) |
| **Methods: Monitoring** | | |
| Data monitoring | 21a | Composition of data monitoring committee (DMC); summary of its role and reporting structure; statement of whether it is independent from the sponsor and competing interests; and reference to where further details about its charter can be found, if not in the protocol. Alternatively, an explanation of why a DMC is not needed |
|  | 21b | Description of any interim analyses and stopping guidelines, including who will have access to these interim results and make the final decision to terminate the trial |
| Harms | 22 | Plans for collecting, assessing, reporting, and managing solicited and spontaneously reported adverse events and other unintended effects of trial interventions or trial conduct |
| Auditing | 23 | Frequency and procedures for auditing trial conduct, if any, and whether the process will be independent from investigators and the sponsor |
| Ethics and dissemination | | |
| Research ethics approval | 24 | Plans for seeking research ethics committee/institutional review board (REC/IRB) approval |
| Protocol amendments | 25 | Plans for communicating important protocol modifications (eg, changes to eligibility criteria, outcomes, analyses) to relevant parties (eg, investigators, REC/IRBs, trial participants, trial registries, journals, regulators) |
| Consent or assent | 26a | Who will obtain informed consent or assent from potential trial participants or authorised surrogates, and how (see Item 32) |
|  | 26b | Additional consent provisions for collection and use of participant data and biological specimens in ancillary studies, if applicable |
| Confidentiality | 27 | How personal information about potential and enrolled participants will be collected, shared, and maintained in order to protect confidentiality before, during, and after the trial |
| Declaration of interests | 28 | Financial and other competing interests for principal investigators for the overall trial and each study site |
| Access to data | 29 | Statement of who will have access to the final trial dataset, and disclosure of contractual agreements that limit such access for investigators |
| Ancillary and post-trial care | 30 | Provisions, if any, for ancillary and post-trial care, and for compensation to those who suffer harm from trial participation |
| Dissemination policy | 31a | Plans for investigators and sponsor to communicate trial results to participants, healthcare professionals, the public, and other relevant groups (eg, via publication, reporting in results databases, or other data sharing arrangements), including any publication restrictions |
|  | 31b | Authorship eligibility guidelines and any intended use of professional writers |
|  | 31c | Plans, if any, for granting public access to the full protocol, participant-level dataset, and statistical code |
| Appendices |  |  |
| Informed consent materials | 32 | Model consent form and other related documentation given to participants and authorised surrogates |
| Biological specimens | 33 | Plans for collection, laboratory evaluation, and storage of biological specimens for genetic or molecular analysis in the current trial and for future use in ancillary studies, if applicable |

*It is strongly recommended that this checklist be read in conjunction with the SPIRIT 2013 Explanation & Elaboration for important clarification on the items. Amendments to the protocol should be tracked and dated. The SPIRIT checklist is copyrighted by the SPIRIT Group under the Creative Commons “[Attribution-NonCommercial-NoDerivs 3.0 Unported](http://www.creativecommons.org/licenses/by-nc-nd/3.0/)” license.

**Additional file 2: prognostic model PATCH**

**PATCH calculator**

(**P**redicting **A**dherence in pa**T**ients with **CH**ronic diseases)

**What is PATCH?**

PATCH is a prognostic and treatment benefit tool that helps healthcare providers to decide if their patient with COPD needs more or less support during pulmonary rehabilitation (PR), by using the probability of adherence. If a patient needs more support, examples of psychosocial interventions that might be helpful are offered.

The prevalence of chronic diseases is rising across Europe, triggered by increasing life expectancy and changing lifestyles (1). The resulting pressures on health systems to address chronic diseases, including chronic obstructive pulmonary disease (COPD), have become a concern for policymakers and -providers (2). One of the possible solutions to keep care affordable is ‘self-management’. The risk of self-management is that it comes at the expense of the effectiveness of PR due to non-adherence.

PATCH answers the questions:

- What is the probability of your patient being adherent?
- Can you responsibly allow your patients with COPD to exercise independently?
- What psychosocial interventions might be used if necessary?

**Explanation**:

1. Use the questionnaires to objectify the independent predictors:

- MRC-score; MRC dyspnea scale
- Depression; 4DSQ depression items
- Intention; four intention items from Ajzen
- Alliance; Work Inventory Assessment (WIA)

1. Enter the scores of each questionnaire in the calculator
2. Result of the calculator is the probability of adherence

**Cut-off value**

The optimal cut-off point of PATCH is based on maximizing the sum of sensitivity and specificity. Based on the cohort of this study a threshold of 53.5% is suggested as the optimal cut-off value to define adherent patients.

Patients with a probability score of ≥ 53.5% are adherent and might be capable of more self-management and need less support from a healthcare provider. However, it is advised to monitor whether the patient remains adherent and health outcomes remain stable.

Patients with a probability of < 53.5% have a (high) risk of non-adherence. In consultation with the patient, one of the psychosocial interventions listed below, in addition to the current intervention, may be used to improve adherence.

**Interventions**

*Teach Back*

Teach-back involves asking patients to explain in their own words what a healthcare provider just has told them. Any misunderstanding is then clarified by the healthcare provider and understanding is checked again. This process continues until the patient can correctly recall the information that was given (3).

*Motivational Interviewing*

Motivational interviewing (MI) is a patient-centred conversational approach to behaviour change. The aim for healthcare providers is to help guide a conversation about change, and activating patients’ intrinsic motivation. To apply this strategy three dimensions can be assessed: 1. Importance: How important is it to the patient to become more physically active? 2. Confidence: How confident does the patient feel that he/she can be more physically active? and 3. Readiness: How much does the patient feel that he/she can start the change process now? (4).

*Cognitive behavioural therapy*

Within Cognitive Behavioural Therapy (CBT) patient and healthcare provider work collaboratively to 1. identify maladaptive thoughts, beliefs and impact of current symptoms or feelings; 2. develop skills to identify, monitor and counteract problematic cognitions related to the specific symptom; and 3. build a repertoire of coping skills appropriate to the target thoughts, beliefs, and behaviours (5).

Since pulmonary rehabilitation (PR) is often offered in group form, there may be added value in offering CBT to the group as well. Three goals can be set here: 1. encourage patients to establish realistic exercise goals that incorporate their personal situation; 2. demonstrate techniques in the group that would help patients reach their goals; and 3. use the group as a way to highlight the common struggle of remaining active (6).

**References**

1. Jakab M, Farrington J, Borgermans L, Mantingh F. Health system respond to noncommunicable diseases: time for ambition. Denmark: WHO Regional Office for Europe; 2018.

2. Winkelmann J, Williams GA, Rijken M, Polin K, Maier CB. Chronic conditions and multimorbidity: skill-mix innovations for enhanced quality and coordination of care. In: Maier CB, Kroezen M, Wismar M, Busse R, editors. Skill-mix Innovation, Effectiveness and Implementation: Improving Primary and Chronic Care. European Observatory on Health Systems and Policies. Cambridge: Cambridge University Press; 2022. p. 152-220.

3. Talevski J, Wong Shee A, Rasmussen B, Kemp G, Beauchamp A. Teach-back: A systematic review of implementation and impacts. PLoS One. 2020;15(4):e0231350.

4. Stonerock GL, Blumenthal JA. Role of Counseling to Promote Adherence in Healthy Lifestyle Medicine: Strategies to Improve Exercise Adherence and Enhance Physical Activity. Prog Cardiovasc Dis. 2017;59(5):455-62.

5. Williams MT, Johnston KN, Paquet C. Cognitive Behavioral Therapy for People with Chronic Obstructive Pulmonary Disease: Rapid Review. Int J Chron Obstruct Pulmon Dis. 2020;15:903-19.

6. Herning MM, Cook JHJ, Schneider JK. Cognitive Behavioral Therapy to Promote Exercise Behavior in Older Adults: Implications for Physical Therapists. Journal of Geriatric Physical Therapy. 2005;28(2):34-8.
